# Hypertension modifies the associations of body mass index and waist circumference with all-cause mortality among older Chinese: a retrospective cohort study

**DOI:** 10.1186/s12877-022-03057-9

**Published:** 2022-05-19

**Authors:** Kaizhi Bai, Xuejiao Chen, Zhan Shi, Kun He, Xueqi Hu, Rui Song, Wenlong Shi, Qingfeng Tian, Songhe Shi

**Affiliations:** 1 College of Public Health, Zhengzhou University, Zhengzhou, Henan China; 2 Department of Pharmacy, People’s Hospital of Zhengzhou, Zhengzhou, Henan China

**Keywords:** Body mass index, Waist circumference, Hypertension, Cohort study, Mortality

## Abstract

**Background:**

The effect of baseline hypertension status on the BMI–mortality association is still unclear. We aimed to explore the relationships of body mass index (BMI) and waist circumference (WC) with all-cause mortality among older hypertensive and normotensive Chinese individuals.

**Methods:**

This retrospective cohort study was conducted in Xinzheng, Henan Province, Central China. The data came from the residents’ electronic health records of the Xinzheng Hospital Information System. A total of 77,295 participants (41,357 hypertensive participants and 35,938 normotensive participants) aged ≥ 60 years were included from January 2011 to November 2019. Cox proportional hazard regression model was used to examine the relationships.

**Results:**

During a mean follow-up of 5.3 years, 10,755 deaths were identified (6,377 in hypertensive participants and 4,378 in normotensive participants). In adjusted models, compared with a BMI of 18.5–24 kg/m^2^, the hazard ratios (HRs) and corresponding 95% confidence intervals (CIs) of BMI < 18.5, 24–28 and ≥ 28 kg/m^2^ for mortality in hypertensive participants were 1.074 (0.927–1.244), 0.881 (0.834–0.931) and 0.856 (0.790–0.929), respectively, and 1.444 (1.267–1.646), 0.884 (0.822–0.949) and 0.912 (0.792–1.051), respectively, in normotensive participants. Compared with normal waist circumference, the adjusted HRs and 95% CIs of central obesity for mortality were 0.880 (0.832–0.931) in hypertensive participants and 0.918 (0.846–0.996) in normotensive participants. A sensitivity analysis showed similar associations for both hypertensive and normotensive participants.

**Conclusion:**

Low BMI and WC were associated with a higher risk of all-cause mortality regardless of hypertension status in older Chinese individuals. The lowest risk of death associated with BMI was in the overweight group in normotensive participants and in the obesity group in hypertensive participants.

**Supplementary Information:**

The online version contains supplementary material available at 10.1186/s12877-022-03057-9.

## Background

In recent years, the proportion of people with a BMI of 25 or greater has been rising substantially worldwide [1]. The prevalence of overweight (25 kg/m^2^ ≤ BMI < 30 kg/m^2^) and obesity (BMI ≥ 30 kg/m^2^) among Chinese adults was 28.1% and 5.2%, respectively [2]. The prevalence of central obesity [waist circumference (WC) ≥ 90 in males and ≥ 85 in females] in Chinese adults was 29.1% (28.6% in males and 29.6% in females), and the estimated number was 277.8 million (140.1 million males and 137.7 females) [3]. Higher BMI or WC is associated with multiple chronic condition, such as hypertension and diabetes [4–6]. However, several studies have shown that overweight participants and even participants with obesity had a lower mortality risk among cardiovascular patients, which is known as the obesity paradox [7, 8]. In addition to cardiovascular patients, the obesity paradox was also found in patients with chronic condition such as chronic kidney disease [9], diabetes [10] and stroke [11]. This finding suggests that the optimal BMI range to maintain health may be different in patients with and without certain diseases. A study in southern China showed that a higher BMI was associated with a lower risk of mortality among hypertensive adults aged 45–75 years, with the lowest risk in those considered obese (Chinese classification, BMI ≥ 28.0 kg/m^2^) [12]. Another study organized in Beijing, China, which examined BMI and all-cause mortality, showed that the lowest risk of all-cause mortality was among hypertensive participants aged 40 to 91 years with a BMI of 24–26 kg/m^2^ [13]. However, those studies did not explore these relationships in normotensive participants, and abdominal adipose tissue measured by WC, which is more metabolically active and is a determinant of metabolic abnormalities of obesity-related disease [3], was not examined. Therefore, this study recruited older adults in Xinzheng, Henan, China, to explore the relationships of WC and BMI with the risk of all-cause mortality among hypertensive participants and normotensive participants.

## Methods

### Participants

**Fig. 1:**
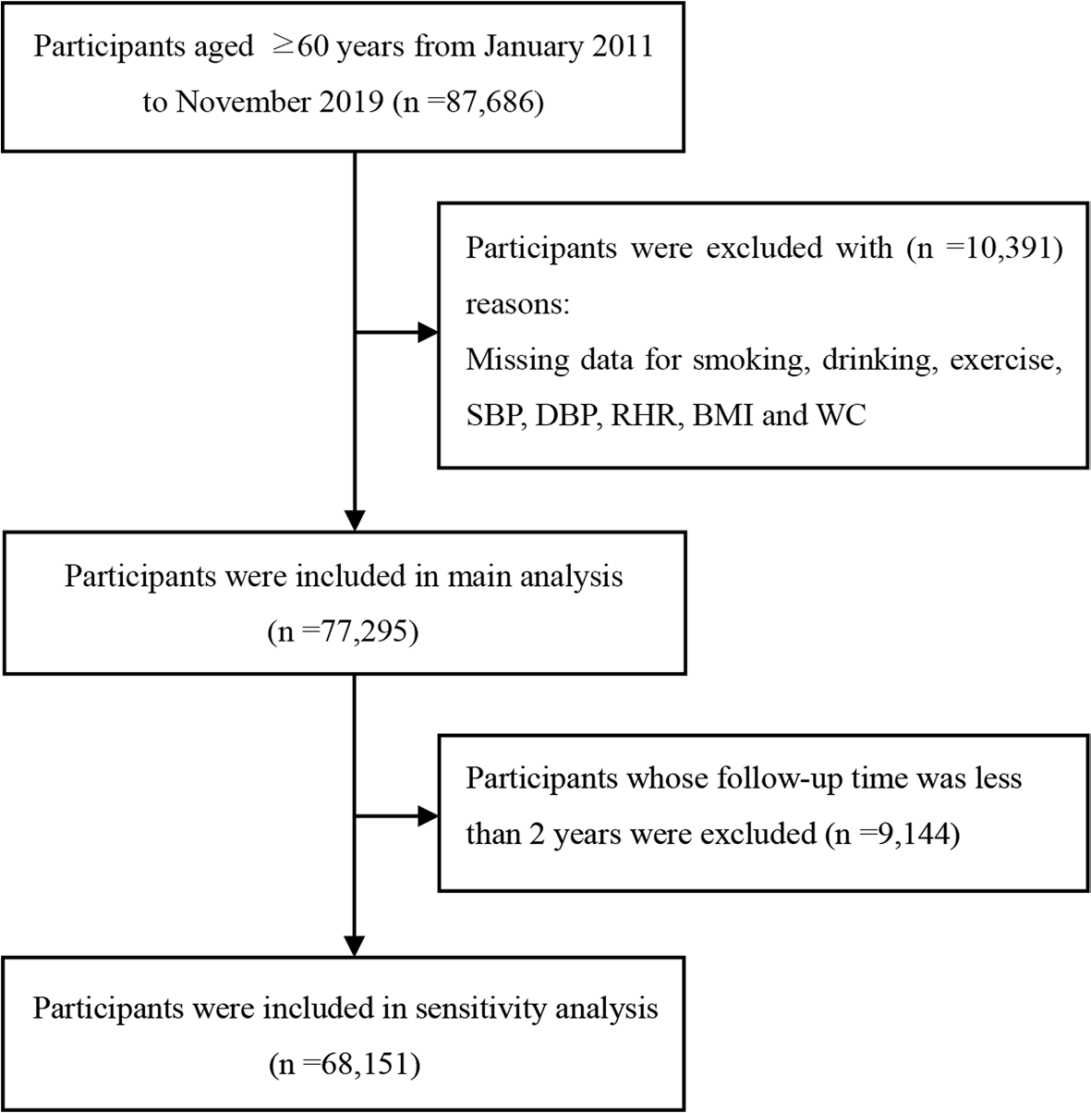
Screening flowchart of participants

The subjects of this study were older people in Xinzheng, Henan Province, Central China. The data came from the residents' electronic health records of the Xinzheng Hospital Information System from January 2011 to November 2019. Doctors set up health records for each resident at their first hospital visit or health examination, and participants aged 60 or older could receive free annual health examinations. At the start of the study, 87,686 older adults were eligible for the study. We excluded participants with the following conditions: (1) missing information for drinking, smoking, exercise, resting heart rate (RHR), systolic blood pressure (SBP) or diastolic blood pressure (DBP) (*n* = 3,447); (2) missing information for WC or BMI (*n* = 6.944). In the end, the study included 77,295 participants [including 41,357 hypertensive participants (30,091 participants self-reported high blood pressure and the percentage is 72.8%) and 35,938 normotensive participants]. A total of 9,144 participants who were followed for less than 2 years were then excluded to execute a sensitivity analysis. The data screening flow chart is presented in Fig. 1. Informed consent was obtained from the subjects, and this study was approved by the Ethics Committee of Zhengzhou University (Reference Number: ZZUIRB2019-019).

### Data collection

Demographic and clinical information was collected at each health checkup for participants. Demographic information included sex (male/female), age, marital status, current drinking (yes/no), current smoking (yes/no), and regular exercise (yes/no). Marital status was defined as married or not, including unmarried, widowed and divorced. Current smoking was defined as having smoked more than 100 cigarettes during their life and still smoking now. Current drinking was defined as drinking occasionally, often or daily compared to never drinking. The definition of regular exercise was 30 min of moderate-intensity exercise more than three times per week. Clinical data included anthropometric measurements, laboratory investigations, and self-reported disease history. Participants wearing light clothing took off their shoes, and then their weight and height were gauged. BMI was calculated as the weight in kilograms divided by the square of the height in meters. BMI was analyzed by the following 3 methods: 1) as four groups according to the BMI classification standard for Chinese individuals: underweight (BMI < 18.5 kg/m^2^), normal (18.5–24 kg/m^2^), overweight (24–28 kg/m^2^), and obesity (≥ 28 kg/m^2^) [14], 2) as eight groups (< 18, 18–20, 20–22, 22–24, 24–26, 26–28, 28–30 and ≥ 30 kg/m^2^), and 3) as a continuous variable. WC was measured at the midpoint of the distance between the lowest costal ridge and the upper border of the iliac crest and was analyzed in the following 2 ways: 1) as a binary variable (normal waist: < 85 cm in females and < 90 cm in males; central obesity: ≥ 85 cm in females and ≥ 90 in males) [15] and 2) as a continuous variable. After participants fasted for 8 h, blood samples were collected to measure blood lipids and blood sugar. After sitting for at least five minutes at rest, the SBP, DBP and radial pulse rate of the participants were measured twice by an electronic sphygmomanometer (Omron HEM-7125, Kyoto, Japan), and the mean value was recorded as the final result.

### Outcome definition

The outcome was all-cause death from January 2011 to November 2019. Information on deaths came from the Centers for Disease Control’s cause of death reporting system in Xinzheng.

### Statistical analysis

Continuous variables were described as means and standard deviations (SDs). Categorical variables were presented as numbers and proportions. The chi-square test for categorical variables and the t-test for continuous variables were used to compare the difference between two groups defined by blood pressure. The associations of BMI and WC with all-cause mortality among normotensive or hypertensive participants were analyzed by a Cox proportional hazard regression model, and HRs with 95% CIs of BMI and WC in categories and continuous variables were expressed in separate models. Model 1 adjusted for age and sex. Model 2 adjusted for confounders including age, sex, marital status, regular exercise, current drinking, current smoking, RHR to estimate the relationship across rising BMI categories. The potentially nonlinear relationship of continuous BMI and WC with all-cause mortality was explored by restricted cubic spline models with four knots. In addition, stratified analysis was performed by subgroups of sex, smoking, alcohol consumption, and exercise status using a Cox regression model to test the consistency of these relationships. Finally, the combined effect of BMI (≥ 24 kg/m^2^ or < 24 kg/m^2^) and WC (≥ 90 cm in males/ ≥ 85 cm in females or < 90 cm in males/ < 85 cm in females) was explored. The proportional hazards assumption was verified with graphical methods and with models including time-by-covariate interactions. Statistical analyses were performed using SPSS V 21 and R V 4.0.3. *P* < 0.05 with two-sided tests was considered statistically significant.

## Results

Hypertensive participants were older and had higher BMI, WC and RHR than normotensive participants. The proportion of female subjects was higher in participants with hypertension (Table 1). The mean (standard deviation, SD) age of the participants was 68.2 (7.4) years, and 52.6% of the participants were females. The correlation coefficient between BMI and WC was 0.695 (*P* < 0.001). The mean (standard deviation, SD) follow-up was 5.3 (2.3) years. In 41,357 hypertensive participants, during 229,630 person-years of follow-up, 6,377 (15.4%) all-cause deaths occurred. In 35,938 normotensive participants, during 178,735 person-years of follow-up, 4,378 (12.2%) all-cause deaths occurred.

**Table 1:** Baseline characteristics of the included participants according to blood pressure status

	**Total**	**Blood pressure status**		***P* Value**
		**Normotension**	**Hypertension**	
Number of participants, n (%)	77,295 (100.0)	35,938 (46.5)	41,357 (53.5)	
Age(years), mean (SD)	68.2 (7.4)	67.7 (7.5)	68.6 (7.3)	< 0.001
BMI, mean (SD)	24.2 (3.3)	23.6 (3.0)	24.8 (3.4)	< 0.001
WC, mean (SD)	82.7 (9.6)	81.3 (9.0)	83.9 (10.0)	< 0.001
Female, n (%)	40,619 (52.6)	18,093 (50.3)	22,526 (54.5)	< 0.001
Married, n (%)	60,825 (78.7)	28,622 (79.6)	32,203 (77.9)	< 0.001
Current smokers, n (%)	9603 (12.4)	4393 (12.2)	5210 (12.6)	0.118
Current drinking, n (%)	5633 (7.3)	2182 (6.1)	3451 (8.3)	< 0.001
Regular exercise, n (%)	14,374 (18.6)	6020 (16.8)	8354 (20.2)	< 0.001
RHR, beats/min, mean (SD)	74.3 (8.2)	74.0 (7.8)	74.6 (8.5)	< 0.001

*Abbreviations*: *BMI* Body mass index, *SD* Standard deviation, *WC* Waist circumference, *RHR* Resting heart rate

The associations of BMI and WC with all-cause mortality are presented in Table 2. For hypertensive participants, the death rate decreased with increasing BMI groups and was lower in participants with central obesity. The normotensive participants experienced similar trends. In hypertensive participants, after adjusting for other covariates, each SD increase in BMI was associated with an 6.5% (5.2%-8.8%) lower risk of all-cause mortality, and compared with BMI 22–24 kg/m^2^, the HRs (95% CIs) of BMI < 18, 18–20, 20–22, 24–26, 26–28, 28–30 and ≥ 30 kg/m^2^ for all-cause mortality were 1.099 (0.913–1.323), 1.145 (1.036–1.267), 1.092 (1.014–1.175), 0.932 (0.868–1.002), 0.902 (0.830–0.980), 0.864 (0.775–0.963) and 0.935 (0.831–1.053), respectively. Each SD increase in WC was associated with a 5.4% (3.1%-7.8%) lower risk of all-cause mortality in Model 2. As a binary variable, the HR (95% CI) of central obesity in the risk of all-cause mortality was 0.880 (0.832–0.931) compared with normal waist.In normotensive participants, after adjusting for other covariates, the HRs (95% CIs) of BMI < 18, 18–20, 20–22, 24–26, 26–28, 28–30 and ≥ 30 kg/m2 for the risk of all-cause mortality were 1.516 (1.287–1.785), 1.278 (1.153–1.416), 1.084 (1.001–1.174), 0.992 (0.906–1.085), 0.824 (0.729–0.932), 0.992 (0.833–1.181) and 0.915 (0.718–1.166), respectively, compared with a BMI of 22–24 kg/m2. Each SD increase in BMI was associated with a 10.9% (7.7%-13.9%) lower risk of all-cause mortality. Each SD increase in WC was associated with a 5.2% (1.9%-8.4%) lower risk of all-cause mortality in Model 2. As a binary variable, the HR (95% CI) of central obesity in the risk of death was 0.918 (0.846–0.996) compared with normal waist. After excluding subjects with a follow-up of less than two years, a sensitivity analysis was conducted to check the robustness of the associations (Supplementary Table 1), and the results showed similar associations. A stratified analysis was performed by subgroups of sex, smoking, alcohol consumption, and exercise status (Supplementary Table 2). Nearly all subgroups of hypertensive and normotensive participants showed that a higher BMI or WC was associated with a lower risk of all-cause mortality.

**Table 2:** HRs of All-cause mortality according to BMI and WC for participants

Participant with hypertension	Death	Pearson-years of follow-up	Mortality rate, per 10,000 pearson-year	Model 1,HR (95%CI)	Model 2,HR (95%CI)	Participant without hypertension	Death	Pearson-years of follow-up	Mortality rate, per 10,000 pearson-year	Model 1,HR (95%CI)	Model 2,HR (95%CI)
BMI grouped by 2 kg/m^2^						BMI grouped by 2 kg/m^2^					
< 18	120	2332.6	514.4	1.127 (0.936–1.357)	1.099 (0.913–1.323)	< 18	162	2681.9	604.1	1.541 (1.309–1.815)	1.516 (1.287–1.785)
18–20	486	10,898.3	445.9	1.159 (1.049–1.282)	1.145 (1.036–1.267)	18–20	501	12,319.9	406.7	1.276 (1.151–1.414)	1.278 (1.153–1.416)
20–22	1159	31,095.6	372.7	1.097 (1.018–1.180)	1.092 (1.014–1.175)	20–22	1072	35,521.0	301.8	1.089 (1.006–1.179)	1.084 (1.001–1.174)
22–24	1825	61,725.7	295.7	Reference	Reference	22–24	1396	58,620.5	238.1	Reference	Reference
24–26	1262	51,445.5	245.3	0.930 (0.866–1.000)	0.932 (0.868–1.002)	24–26	726	36,923.7	196.6	0.984 (0.899–1.076)	0.992 (0.906–1.085)
26–28	803	37,189.9	215.9	0.896 (0.824–0.973)	0.902 (0.830–0.980)	26–28	312	20,026.7	155.8	0.814 (0.720–0.921)	0.824 (0.729–0.932)
28–30	396	19,245.8	205.8	0.860 (0.771–0.959)	0.864 (0.775–0.963)	28–30	140	8176.0	171.2	0.989 (0.830–1.177)	0.992 (0.833–1.181)
≥ 30	326	15,696.9	207.7	0.938 (0.833–1.056)	0.935 (0.831–1.053)	≥ 30	69	4465.2	154.5	0.911 (0.715–1.161)	0.915 (0.718–1.166)
BMI, kg/m^2^						BMI, kg/m^2^					
< 18.5	190.0	3879.5	489.8	1.053 (0.908–1.221)	1.074 (0.927–1.244)	< 18.5	246	4394.1	559.8	1.459 (1.280–1.663)	1.444 (1.267–1.646)
18.5–24	3400.0	102,172.8	332.8	Reference	Reference	18.5–24	2885	104,749.2	275.4	Reference	Reference
24–28	2065.0	88,635.4	233.0	0.877 (0.823–0.933)	0.881 (0.834–0.931)	24–28	1038	56,950.4	182.3	0.874 (0.814–0.939)	0.884 (0.822–0.949)
≥ 28	722.0	34,942.6	206.6	0.860 (0.766–0.966)	0.856 (0.790–0.929)	≥ 28	209	12,641.1	165.3	0.908 (0.788–1.046)	0.912 (0.792–1.051)
BMI as a continuous variable (per SD increase)	6377	229,630.3	277.7	0.930 (0.906–0.953)	0.935 (0.912–0.958)	BMI as a continuous variable (per SD increase)	4378	178,734.8	244.9	0.887 (0.856–0.918)	0.891 (0.861–0.923)
WC, cm						WC, cm					
< 90 (male)/ < 85 (female)	4693	148,868.7	315.2	reference	reference	< 90 (male)/ < 85 (female)	3676	136,719.9	268.9	Reference	Reference
≥ 90 (male)/ ≥ 85 (female)	1684	80,761.6	208.5	0.873 (0.825–0.923)	0.880 (0.832–0.931)	≥ 90 (male)/ ≥ 85 (female)	702	42,014.9	167.1	0.909 (0.838–0.986)	0.918 (0.846–0.996)
WC as a continuous variable (per SD increase)	6377	229,630.3	277.7	0.941 (0.918–0.965)	0.946 (0.922–0.969)	WC as a continuous variable (per SD increase)	4378	178,734.8	244.9	0.944 (0.912–0.976)	0.948 (0.916–0.981)

*Abbreviations*: *HR* Hazard ratio, *CI* Confidential interval, *BMI* Body mass index, *WC* Waist circumference

Model 1: Adjusted age and sex

Model 2: Model 1 plus marital status, current drinking, current smoking, regular exercise, resting heart rate

**Fig. 2:**
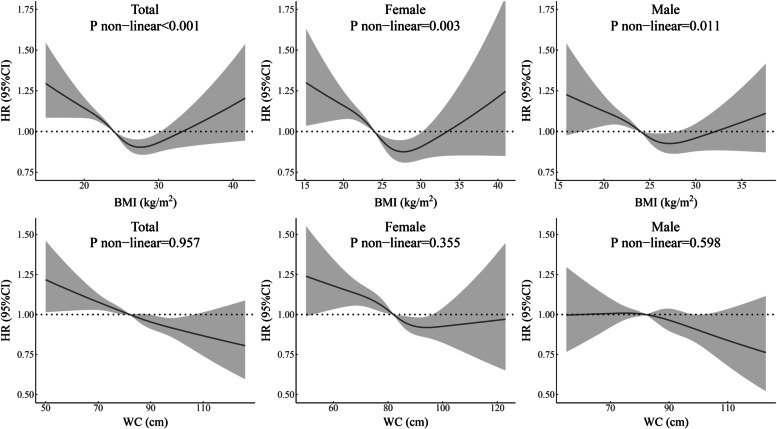
Relationship of BMI and WC with the risk of all-cause mortality for all hypertensive participants and subgroups of males and females. HRs are adjusted for age, sex (not for sex subgroup analysis), marital status, current drinking, current smoking, regular exercise, resting heart rate. Abbreviations: *BMI* body mass index; *CI* confidential interval; *HR* hazard ratio; *WC* waist circumference

BMI had a nonlinear association with all-cause mortality among all hypertensive participants and female and male subgroups based on the adjusted Cox model (Fig. 2). These associations all showed a reverse J-shaped curve. The association between WC and death indicated that the risk was highest when WC values were very low among all participants and the female subgroup with hypertension.

**Fig. 3:**
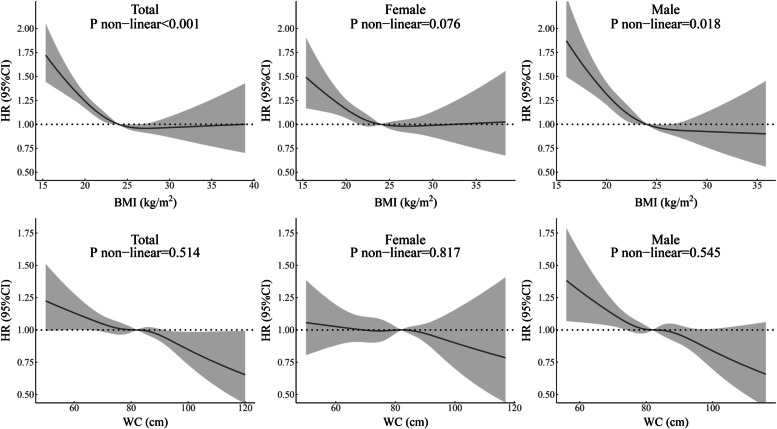
Relationship of BMI and WC with the risk of all-cause mortality for all normotensive participants and subgroups of males and females. HRs are adjusted for age, sex (not for sex subgroup analysis), marital status, current drinking, current smoking, regular exercise, resting heart rate. Abbreviations: *BMI* body mass index; *CI* confidential interval; *HR* hazard ratio; *WC* waist circumference

BMI had a nonlinear association with all-cause mortality among all normotensive participants and male subgroups, and the risk increased among those with very low BMI values (Fig. 3). The female subgroup had a similar curve with no significant nonlinear association. Lower WC values were associated with a higher death risk for all normotensive participants and the male subgroup, and this relationship was not found in the female subgroup.

**Fig. 4:**
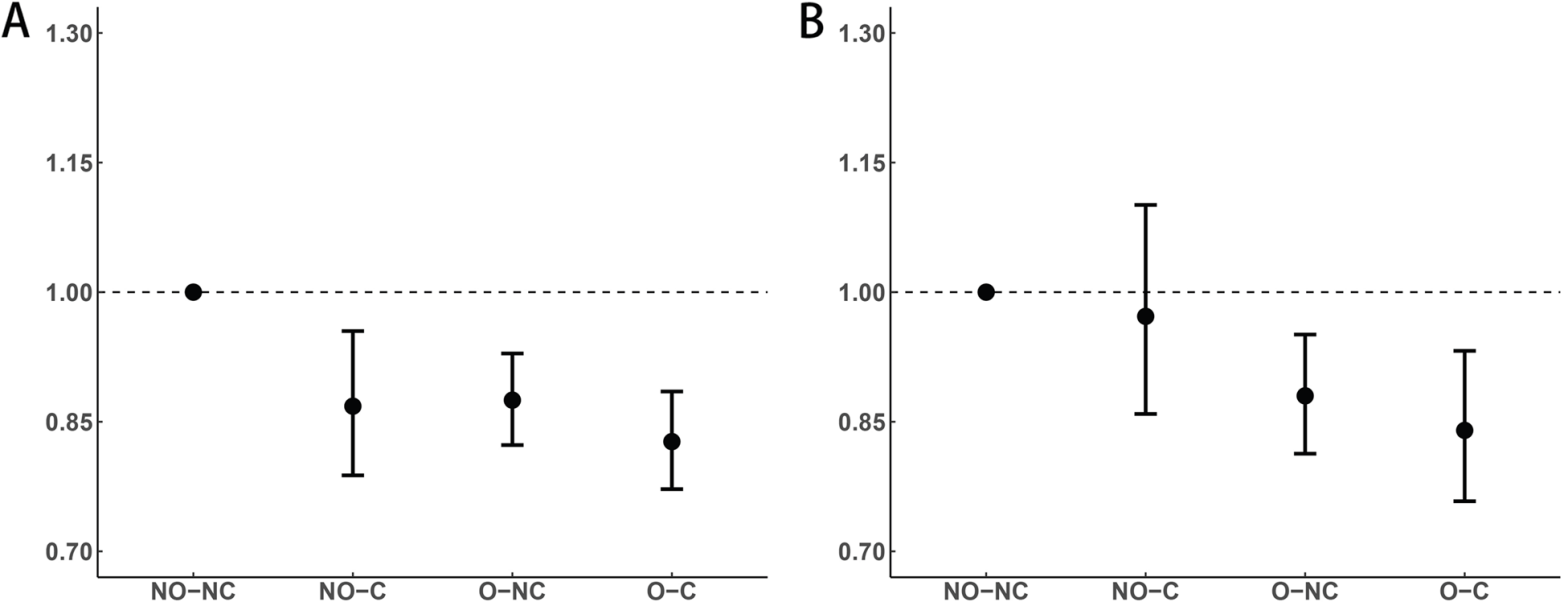
Combined effect of BMI and WC with all-cause mortality for hypertensive (**A**) and normotensive (**B**) participants. HRs adjusted for age, sex, marital status, current drinking, current smoking, regular exercise, resting heart rate. Abbreviations: *NO* non-overweight (BMI ≤ 23 kg/m2); *O* overweight (BMI ≥ 23 kg/m2); *NC* non-central obesity (WC < 90 in male/WC < 85 in female); *C* central obesity (WC ≥ 90 in male/WC ≥ 85 in female); *CI* confidential interval; *HR* hazard ratio

The combined effect of BMI and WC on all-cause mortality in hypertensive and normotensive participants is shown in Fig. 4. For hypertensive participants, after adjusting for other covariates, the HRs (95% CIs) for all-cause mortality of the nonoverweight and central obesity, overweight and noncentral obesity, and overweight and central obesity groups were 0.868 (0.788–0.955), 0.875 (0.823–0.929) and 0.827 (0.772–0.885), respectively, compared with the nonoverweight and noncentral obesity groups. For normotensive participants, the HRs (95% CIs) for all-cause mortality of the nonoverweight and central obesity, overweight and noncentral obesity, and overweight and central obesity groups were 0.972 (0.859–1.101), 0.880 (0.813–0.951), and 0.840 (0.758–0.932), respectively, compared with the nonoverweight and noncentral obesity group.

## Discussion

In this study, we found that compared with normal weight, the underweight group was associated with a higher risk of all-cause mortality, overweight was associated with a lower risk of death in normotensive participants, and overweight and obesity were associated with a lower risk of death in hypertensive participants. The nadir of risk is BMI 26–28 kg/m^2^ in normotensive participants and BMI 28–30 kg/m^2^ in hypertensive participants, according to eight groups of BMI models. Reverse J-shaped curves were discovered in hypertensive participants, but the curves were almost horizontal with a BMI of ≥ 25 in normotensive participants. The risk of mortality decreased with an increase in WC regardless of hypertension status, and the protective effect of central obesity against death in hypertensive participants was higher than that in normotensive participants.

Our results found that the lowest risk of mortality in the BMI group was for a BMI of 26–28 kg/m^2^ in normotensive participants, similar to the geographically distant USA in NHANES [16, 17]. A prospective cohort study [18] that had a similar result to ours enrolled participants from all 30 provinces in mainland China and found that the lowest risk of mortality in the BMI group was for a BMI of 25–26.9 kg/m^2^ in participants aged ≥ 65 and 24.0–24.9 kg/m^2^ in participants aged < 65. A cohort study conducted in Taipei [19] found that the lowest risk of mortality in the BMI group was for a BMI of 30–34.9 kg/m^2^. A Korean study [20] found that the fourth quartile of BMI had the lowest risk of death. Some studies [21–23] have found that the risk of death relating to BMI was lower compared to our study, which may be due to the difference in the age range of the participants for the following reasons. Adipose tissue stores energy and nutrients to prevent malnutrition, maintain bone mineral density and prevent osteoporotic fractures in older adults and can also provide a cushion in the event of an impact, preventing fractures in falls [24]. Overweight and obese older adults may have better antioxidant defenses [21]. Adipose tissue in older adults provides protection against death and complications from cardiovascular disease (CVD) and non-CVD diseases, and this effect is stronger than the influence of adipose tissue on death and disease [25]. A study conducted in Taiwan suggested a different conclusion: the BMI range with the lowest risk was 24–25.9 kg/m^2^ in both subgroups aged ≥ 65 and < 65. This difference may be because the study participants were mainly volunteers, which causes a bias. A meta-analysis conducted in older white adults found that the lowest risk of death was at a BMI of 27–27.9 kg/m^2^, which is higher than that in younger people [26].

Meanwhile, we found that the lowest risk in BMI in hypertensive participants was 28–30 kg/m^2^, which was higher than the 26–28 kg/m^2^ in hypertensive participants; obesity in hypertensive participants had a significant protective effect, which was not found in normotensive participants. Several studies that focused on the BMI-mortality relationship in hypertensive participants concluded similar results. Due to the high correlation between BMI and WC, we found a kind of waist paradox, the protective effect of central obesity against death in hypertensive participants was higher than that in normotensive participants.

In a cohort study in Jiangsu and Anhui [12], Yang et al. found that compared with normal weight, the risk of death was lower in overweight and obese individuals, and the reverse J-shaped curve also appeared when the interval between groups was reduced. Using electronic health records [27], Wang et al. also found a reverse J-shaped relationship between BMI and all-cause mortality. In studies of BMI and all-cause mortality by Sun et al. [28] and Chung et al. [29], the reverse J-shaped relationship was discovered in the subgroup analysis of patients with hypertension. In a study of the obesity paradox among subjects with both hypertension and coronary artery disease [30], Uretsky et al. found the existence of this paradox, but this study did not adjust for other confounded covariates. A meta-analysis including 14 studies also found a reverse J-shaped relationship in all-cause mortality among hypertensive participants.

This phenomenon may be due to the following reasons. First, obese people were more willing to check their own physical condition, so diseases could be found and treated earlier, and they also have better compliance with doctors' treatment plans [31, 32]. Second, reverse causality exists in observational studies, and people may lose weight after illness and before death [12, 29], which results in participants with overweight, obesity and central obesity having a lower risk of all-cause mortality. Third, some studies suggest that people with obesity might have a higher socioeconomic status, which may explain this result, but some studies also suggested that the link between obese people and higher socioeconomic status exists only in some poor countries [33]. Some studies have shown that the influence of socioeconomic status and education level on Chinese individuals is different by sex, and the results are heterogeneous [34–36]. Therefore, it is difficult to explain this result by the socioeconomic status of people with obesity. Fourth, some studies showed that overweight and obese people have a lower risk of depression, and this inverse correlation was stronger in older individuals than in younger people [37, 38]. Fourth, patients with obesity may have lower plasma renin activity and systemic vascular resistance [39], and drug administration may play an important role in this result; adipose tissue releases hormones that have anti-inflammatory effects [20].

Some studies produced different results. For example, Li et al. [13] found that the risk of all-cause death was higher in individuals with a BMI of < 22 and ≥ 30 kg/m^2^ relative to those with a BMI of 24–26 kg/m^2^, which presented a U-shaped curve in hypertensive people. This may be because of the small sample size of this study, with a total of 2535 subjects enrolled. In contrast to our findings, McAuley et al. [40] found that, compared with a BMI of 18.5–24.9 kg/m^2^, 25.0–29.9 kg/m^2^ did not have a statistically significant effect on death. The potential reason may be that the study was underrepresented, as most of the participants came from an upper or middle social status and excluded those whose BMI was < 18.5 kg/m^2^.

Some studies have shown that poor antihypertensive medication adherence leads to a higher risk of death [41, 42]. The obesity paradox among hypertensive people may be due to better medication compliance among obese individuals. A study of the Avoiding Cardiovascular Events through Combination Therapy in Patients Living with Systolic Hypertension (ACCOMPLISH) trial has shown that there was an obesity paradox in participants taking benazepril and hydrochlorothiazide, compared with those taking benazepril and amlodipine. That’s because benazepril and hydrochlorothiazide were not as effective as benazepril and amlodipine in participants with lower BMI. This indicates that people with different adipose tissues may be suitable for different treatment methods for hypertension, and non-obese individuals may not be suitable for thiazide-like diuretic treatment [43]. So the obesity paradox in hypertension may be due to inappropriate use of antihypertensive medication. Another study found no significant association between BMI and death among hypertensive people taking placebo compared with those taking a blood pressure medication [44]. Unfortunately, this study did not include information of antihypertensive medication.

There are some advantages of our study. First, the data of this cohort study were obtained from a large-scale periodical health check in Henan, China. Demographic and laboratory data were collected, and the sample size and statistical power were adequate. Second, few studies have analyzed WC, which can reflect abdominal adipose tissue, and it was analyzed in our study. Third, height and weight were objectively measured rather than self-reported. Finally, we did not adjusted for SBP, DBP, previous history of diabetes, cancer, coronary heart disease and stroke, which are influenced by excess body fat, so we to some extent avoided the overadjustment problem [45].

However, some limitations of this study should be noted. Because the subjects were all older than 60, these relationships should be generalized cautiously to people younger than 60. We adjusted for confounders including age, sex, marital status, exercise, drinking, smoking, RHR, but some potential factors may exist that we did not adjust for, such as antihypertensive medication.

### Conclusion

Overall, this study found that the lowest risk of death relating to BMI was in the overweight group in normotensive older adults and in the obese group in hypertensive older adults. There was a reverse J-shaped relationship between BMI and all-cause mortality in hypertensive older adults, and in normotensive older adults, the risk of all-cause mortality was higher with a low BMI. Participants with central obesity had a lower risk of all-cause mortality regardless of hypertension status. More research is needed to explore the optimal BMI range for hypertensive and normotensive older people to decrease their risk of death.

## Supplementary Information

**Additional file 1:** **Supplementary Table 1.** Sensitive analysis of BMI and WC with All-cause mortality. **Supplementary Table 2.** Hazard ratios of All-cause mortality according to BMI or WC for various subgroups.

## Data Availability

The datasets generated and/or analysed during the current study are not publicly available due to confidentiality requirements of third parties, but are available from the corresponding author on request.

## Abbreviations

BMI: Body mass index
WC: Waist circumference
HR: Hazard ratio
CI: Confidence interval
RHR: Resting heart rate
SBP: Systolic blood pressure
DBP: Diastolic blood pressure
SD: Standard deviation
CVD: Cardiovascular disease
NO-NC: Nonoverweight and noncentral obesity
NO-C: Nonoverweight and central obesity
O-NC: Overweight and noncentral obesity
O-C: Overweight and central obesity
