# Supplementary material for: Hypertension modifies the associations of body mass index and waist circumference with all-cause mortality among older Chinese: a retrospective cohort study
Source: BMC Geriatr. 2022 May 19;22:441. doi: 10.1186/s12877-022-03057-9 (PMC9118767; doi:10.1186/s12877-022-03057-9)
Supplement: Supplementary file 1 — Additional file 1: Supplementary Table 1. Sensitive analysis of BMI and WC with All-cause mortality. Supplementary Table 2. Hazard ratios of All-cause mortality according to BMI or WC for various subgroups. [file 12877_2022_3057_MOESM1_ESM.docx]

**Supplementary Table 1** Sensitive analysis of BMI and WC with All-cause mortality

| **Participant with hypertension** | **Death** | **Pearson-years of follow-up** | **Mortality rate, per 10000 pearson-year** | **Model 1,HR**  **(95%CI)** | **Model 2,HR**  **(95%CI)** | **Participant without hypertension** | **Death** | **Pearson-years of follow-up** | **Mortality rate, per 10000 pearson-year** | **Model 1,HR**  **(95%CI)** | **Model 2,HR**  **(95%CI)** |
| --- | --- | --- | --- | --- | --- | --- | --- | --- | --- | --- | --- |
|  |  |  |  |  |  |  |  |  |  |  |  |
| BMI grouped by 2 kg/m^2^ |  |  |  |  |  | BMI grouped by 2 kg/m^2^ |  |  |  |  |  |
| <18.5 | 111 | 2282.0 | 486.4 | 1.170 (0.964-1.418) | 1.143 (0.942-1.386) | <18.5 | 139 | 2552.3 | 544.6 | 1.507 (1.264-1.798) | 1.481 (1.241-1.766) |
| 18-20 | 443 | 10654.4 | 415.8 | 1.178 (1.060-1.309) | 1.165 (1.048-1.294) | 18-20 | 438 | 11832.5 | 370.2 | 1.247 (1.117-1.392) | 1.248 (1.118-1.392) |
| 20-22 | 1041 | 30417.6 | 342.2 | 1.090 (1.008-1.178) | 1.087 (1.005-1.175) | 20-22 | 956 | 34300.9 | 278.7 | 1.084 (0.997-1.180) | 1.079 (0.992-1.174) |
| 22-24 | 1651 | 60353.8 | 273.6 | Reference | Reference | 22-24 | 1248 | 56523.3 | 220.8 | Reference | Reference |
| 24-26 | 1145 | 50143.0 | 228.3 | 0.930 (0.863-1.003) | 0.933 (0.865-1.006) | 24-26 | 656 | 35465.3 | 185.0 | 0.988 (0.898-1.086) | 0.995 (0.905-1.094) |
| 26-28 | 729 | 36153.3 | 201.6 | 0.896 (0.821-0.978) | 0.903 (0.827-0.985) | 26-28 | 279 | 19168.9 | 145.5 | 0.811 (0.712-0.924) | 0.820 (0.720-0.935) |
| 28-30 | 359 | 18658.6 | 192.4 | 0.859 (0.766-0.964) | 0.865 (0.771-0.970) | 28-30 | 129 | 7817.3 | 165.0 | 1.018 (0.848-1.222) | 1.020 (0.850-1.225) |
| ≥30 | 300 | 15250.1 | 196.7 | 0.950 (0.839-1.075) | 0.949 (0.839-1.074) | ≥30 | 60 | 4246.1 | 141.3 | 0.880 (0.678-1.140) | 0.882 (0.681-1.144) |
| BMI, kg/m^2^ |  |  |  |  |  | BMI, kg/m^2^ |  |  |  |  |  |
| <18.5 | 174 | 3795.4 | 458.4 | 1.118 (0.959,1.303) | 1.110 (0.943,1.282) | <18.5 | 213 | 4178.3 | 509.8 | 1.445 (1.256-1.663) | 1.430 (1.243-1.646) |
| 18.5-24 | 3072 | 99912.4 | 307.5 | Reference | Reference | 18.5-24 | 2568 | 101030.7 | 254.2 | Reference | Reference |
| 24-28 | 1874 | 86296.3 | 217.2 | 0.875 (0.826,0.927) | 0.881 (0.831,0.934) | 24-28 | 935 | 54634.1 | 171.1 | 0.880 (0.816-0.949) | 0.889 (0.825-0.959) |
| ≥28 | 659 | 33908.7 | 194.3 | 0.857 (0.788,0.933) | 0.862 (0.792,0.939) | ≥28 | 189 | 12063.4 | 156.7 | 0.920 (0.793-1.068) | 0.924 (0.796-1.072) |
| BMI as a continuous variable (per SD increase) | 5779 | 223912.8 | 258.1 | 0.928 (0.904,0.953) | 0.932 (0.908,0.957) | BMI as a continuous variable (per SD increase) | 3905 | 171906.6 | 227.2 | 0.892 (0.860-0.926) | 0.897 (0.865-0.931) |
| WC, cm |  |  |  |  |  | WC, cm |  |  |  |  |  |
| <90(male)/<85(female) | 4260 | 145895.8 | 290.0 | Reference | Reference | <90(male)/<85(female) | 3298 | 131967.5 | 249.9 | Reference | Reference |
| ≥90(male)/≥85(female) | 1519 | 78017.0 | 194.7 | 0.873 (0.823,0.926) | 0.880 (0.829,0.934) | ≥90(male)/≥85(female) | 607 | 39939.1 | 152.0 | 0.877 (0.804-0.957) | 0.885 (0.811-0.966) |
| WC as a continuous variable (per SD increase) | 5779 | 223912.8 | 258.1 | 0.943 (0.919,0.968) | 0.948 (0.923,0.973) | WC as a continuous variable (per SD increase) | 3905 | 171906.6 | 227.2 | 0.940 (0.907-0.975) | 0.944 (0.910-0.979) |

Abbreviations: *HR* hazard ratio; *CI* confidential interval; *BMI* body mass index; *WC* waist circumference.

Model 1: Adjusted age and sex.

Model 2: Model 1 plus marital status, current drinking, current smoking, regular exercise, resting heart rate.

**Supplementary Table 2** Hazard ratios of All-cause mortality according to BMI or WC for various subgroups

|  | **Gender HR(95%CI)** | | **Smoking status HR(95%CI)** | | **Drinking status HR(95%CI)** | | **Exercise status HR(95%CI)** | |
| --- | --- | --- | --- | --- | --- | --- | --- | --- |
|  | **Female** | **Male** | **Current smoking** | **No current smoking** | **Current drinking** | **No current drinking** | **Regular exercise** | **No regular exercise** |
| **Participant with hypertension** |  |  |  |  |  |  |  |  |
| BMI, kg/m^2^ |  |  |  |  |  |  |  |  |
| <18.5 | 1.122 (0.932-1.350) | 0.997 (0.783-1.268) | 0.802 (0.500-1.287) | 1.114 (0.955-1.300) | 0.710 (0.350-1.441) | 1.100 (0.947-1.278) | 1.164 (0.757-1.789) | 1.062 (0.908-1.241) |
| 18.5-24 | Reference | Reference | Reference | Reference | Reference | Reference | Reference | Reference |
| 24-28 | 0.869 (0.801-0.943) | 0.894 (0.829-0.963) | 0.862 (0.742-1.002) | 0.883 (0.832-0.937) | 0.865 (0.718-1.042) | 0.882 (0.833-0.935) | 0.831 (0.715-0.966) | 0.891 (0.840-0.946) |
| ≥28 | 0.817 (0.730-0.914) | 0.903 (0.803-1.016) | 0.959 (0.772-1.192) | 0.842 (0.771-0.919) | 0.985 (0.772-1.257) | 0.842 (0.772-0.917) | 0.985 (0.809-1.200) | 0.833 (0.762-0.910) |
| BMI as a continuous variable (per SD increase) | 0.926 (0.895-0.958) | 0.946 (0.912-0.981) | 0.951 (0.889-1.018) | 0.932 (0.908-0.957) | 0.973 (0.895-1.057) | 0.931 (0.907-0.955) | 0.969 (0.907-1.036) | 0.930 (0.906-0.955) |
| WC, cm |  |  |  |  |  |  |  |  |
| <90 (male)/<85 (female) | Reference | Reference | Reference | Reference | Reference | Reference | Reference | Reference |
| ≥90 (male)/≥85 (female) | 0.861 (0.796-0.931) | 0.913 (0.841-0.990) | 0.882 (0.756-1.029) | 0.882 (0.830-0.937) | 0.863 (0.715-1.042) | 0.883 (0.832-0.937) | 0.899 (0.780-1.036) | 0.878 (0.826-0.934) |
| WC as a continuous variable (per SD increase) | 0.934 (0.902-0.966) | 0.966 (0.932-1.002) | 0.901 (0.839-0.967) | 0.954 (0.929-0.979) | 0.891 (0.817-0.972) | 0.952 (0.927-0.977) | 0.918 (0.859-0.981) | 0.951 (0.926-0.977) |
| **Participant without hypertension** |  |  |  |  |  |  |  |  |
| BMI, kg/m^2^ |  |  |  |  |  |  |  |  |
| <18.5 | 1.451 (1.217-1.731) | 1.432 (1.177-1.741) | 1.156 (0.754-1.773) | 1.489 (1.298-1.709) | 1.129 (0.612-2.083) | 1.461 (1.278-1.671) | 1.204 (0.790-1.836) | 1.475 (1.285-1.692) |
| 18.5-24 | Reference | Reference | Reference | Reference | Reference | Reference | Reference | Reference |
| 24-28 | 0.918 (0.825-1.021) | 0.858 (0.778-0.945) | 0.739 (0.591-0.924) | 0.901 (0.836-0.972) | 0.786 (0.572-1.081) | 0.888 (0.825-0.956) | 1.099 (0.906-1.333) | 0.853 (0.789-0.922) |
| ≥28 | 0.956 (0.789-1.158) | 0.882 (0.715-1.088) | 0.659 (0.403-1.077) | 0.941 (0.811-1.091) | 0.762 (0.411-1.415) | 0.918 (0.794-1.062) | 0.723 (0.467-1.119) | 0.945 (0.814-1.098) |
| BMI as a continuous variable (per SD increase) | 0.931 (0.888-0.977) | 0.853 (0.810-0.898) | 0.848 (0.759-0.947) | 0.896 (0.863-0.929) | 0.926 (0.796-1.078) | 0.889 (0.858-0.922) | 0.935 (0.848-1.032) | 0.886 (0.853-0.920) |
| WC, cm |  |  |  |  |  |  |  |  |
| <90 (male)/<85 (female) | Reference | Reference | Reference | Reference | Reference | Reference | Reference | Reference |
| ≥90 (male)/≥85 (female) | 0.960 (0.857-1.075) | 0.878 (0.780-0.989) | 0.781 (0.603-1.011) | 0.933 (0.856-1.017) | 0.806 (0.569-1.141) | 0.926 (0.851-1.007) | 0.944 (0.761-1.171) | 0.915 (0.838-1.000) |
| WC as a continuous variable (per SD increase) | 0.982 (0.936-1.030) | 0.920 (0.876-0.965) | 0.903 (0.811-1.004) | 0.953 (0.919-0.988) | 1.021 (0.881-1.182) | 0.945 (0.912-0.979) | 1.009 (0.916-1.112) | 0.940 (0.907-0.975) |

Abbreviations: *HR* hazard ratio; *CI* confidential interval; *BMI* body mass index; *WC* waist circumference.

Model adjusted age, sex (except for sex subgroups), marital status, current drinking (except for drinking subgroups), current smoking (except for smoking subgroups), regular exercise (except for exercise subgroups), resting heart rate.
